# Fatigue-Mediated Loss of Complexity is Contraction-Type Dependent in Vastus Lateralis Electromyographic Signals

**DOI:** 10.3390/sports7040078

**Published:** 2019-04-02

**Authors:** Luis R. Hernandez, Clayton L. Camic

**Affiliations:** Department of Kinesiology and Physical Education, Northern Illinois University, DeKalb, IL 60115, USA; ccamic1@niu.edu

**Keywords:** quadriceps, sEMG, sample entropy, DFA, eccentric, concentric, isometric

## Abstract

The purpose of this study was to investigate the effect of fatigue status and contraction type on complexity of the surface electromyographic (sEMG) signal. Twelve females (mean age ± SD = 21.1 ± 1.4 years) performed three fatigue-inducing protocols that involved maximal concentric, eccentric, or isometric knee-extensor contractions over three non-consecutive sessions. Pre- and post-fatigue assessments were also completed each session and consisted of three maximal efforts for each type of contraction. Complexity of sEMG signals from the vastus lateralis was assessed using Sample Entropy (SampEn) and Detrended Fluctuation Analysis (DFA) as expressed using the scaling exponent α. The results showed that fatigue decreased (*p* < 0.05) sEMG complexity as indicated by decreased SampEn (non-fatigued: 1.57 ± 0.22 > fatigued: 1.46 ± 0.25) and increased DFA α (non-fatigued: 1.27 ± 0.26 < fatigued: 1.32 ± 0.23). In addition, sEMG complexity was different among contraction types as indicated by SampEn (concentric: 1.58 ± 0.22 > eccentric: 1.47 ± 0.27 and isometric: 1.50 ± 0.21) and DFA α (concentric: 1.27 ± 0.18 < isometric: 1.32 ± 0.18). Thus, these findings suggested sEMG complexity is affected by fatigue status and contraction type, with the degree of fatigue-mediated loss of complexity dependent on the type of contraction used to elicit fatigue.

## 1. Introduction

Physiological systems exhibit high levels of complexity as defined by the presence of an informationally rich variability, the dynamics of which is characterized by non-linearities and long-range temporal correlations (LRTCs), in the structure of their corresponding outputs [1]. This variability reflects a dynamic interplay amongst the many control systems, couplings, and feedback loops operating across a variety of spatiotemporal scales and between various physiological systems to efficiently detect, respond, and adapt to a diverse set of stressors/demands [2,3]. Loss of/low levels of complexity correspond to a variety of sub-optimal states such as disease/disorder [4], injury [5], and fatigue [6]. Recently, complexity has been recognized as a defining feature of healthy physiological functioning [2,7]. In general, it is believed that complexity is dependent on the number of active constituents in a system and the degree of interaction between constituents [1]. As of yet, however, there exists no generally accepted framework to explain the mechanisms underlying complexity for specific physiological systems. Thus, the investigation of factors which influence complexity for specific systems may provide insight to potential mechanisms of regulation, the understanding and manipulation of which could guide diagnostic and rehabilitative efforts in the attempt to steer systems towards optimal levels of complexity and function [2,8].

A variety of tools from the field of non-linear dynamics have been used to assess complexity. Sample entropy (SampEn), a non-linear measure of statistical irregularity [9], is often used as an indirect index of physiological complexity with values typically ranging from 0 to 2. Higher values indicate higher irregularity which reflect more complex dynamics [3]. Conversely, lower values indicate lower irregularity and thus less complex dynamics. Of note, the breakdown of LRTCs in physiological signals can also result in increased irregularity. In this case, the signal is more “random” or noise-like and less informationally rich (i.e., less complex) [1]. Therefore, it is suggested that a variety of statistical measures be used to assess complexity, as opposed to any single measure in particular.

Another method commonly used to assess complexity is Detrended Fluctuation Analysis (DFA). DFA assesses the self-affinity of a signal, yielding a scaling factor (α) which characterizes its correlational features [10]. Briefly, if 0.5 < α < 1, the signal displays persistent power-law correlations characteristic of fractal dynamics [11,12]. If α > 1, correlations exist but are no longer power law in form. Special cases occur when α = 0.5 (white noise), α = 1 (pink noise), and α = 1.5 (brown noise). A breakdown in the correlational features of a signal (α approaches 0.5) is associated with a loss of complexity [1]. However, disordered states have also been observed to show strengthened LRTCs [13]. Thus, it is suggested that optimal levels for the correlational features of a system exist, deviations that result in sub-optimal functioning [14]. Specifically, it is thought that an α of 1 reflects optimal functioning, being an intermediate compromise between the roughness of white noise (α = 0.5) and smoothness of brown noise (α = 1.5) [10].

The use of surface electromyography (sEMG) for recording the electrical activity of skeletal muscle has been used extensively to study neuromuscular function across several disciplines. The sEMG signal is itself highly complex, dynamic, and non-linear in nature [15]. In the context of the neuromuscular system, the constituents are motor units and thus mechanisms that effect the activation and the interactions of which are likely candidates for regulatory mechanisms. Factors related to motor unit activation such as fatigue [16,17,18,19], intensity/load, and velocity [20,21] have been observed to affect the complexity of sEMG signals. These findings suggest a role for motor unit recruitment and discharge rates in neuromuscular complexity, although further research is warranted. Nonetheless, complex dynamics are often the result of a continuous interplay between various mechanisms operating on a variety of scales [3]. As such, it would be preemptive to attribute the complexity of a system to a singular mechanism or factor. Data concerning other potential factors (e.g., type of muscle contraction or fatigue as induced by different contraction types) are limited. Therefore, the purpose of this study was to investigate: (1) The effect of fatigue status on complexity, (2) the effect of contraction type on complexity, and (3) potential differences in the effect of fatigue as induced by different types of contractions. We hypothesized that: (1) sEMG signals recorded during fatigued conditions will be less complex than those during non-fatigued conditions, (2) the complexity of the sEMG signal will be contraction-type dependent, and (3) the degree to which fatigue results in less complex sEMG signals will depend on the type of contraction used to elicit fatigue.

## 2. Materials and Methods

### 2.1. Subjects

Twelve females (mean age ± SD = 21.1 ± 1.4 years, body mass = 63.3 ± 7.4 kg) volunteered to participate in the study. An a priori power analysis using G*Power 3.1 (Universität Düsseldorf, Düsseldorf, Germany) indicated a sample size of 12 was required to achieve power (1-β) of 0.80 with an effect size of 0.3 and alpha of 0.05. All participants were moderately-trained with an average of 2.7 ± 1.7 h resistance training per week and 4.8 ± 2.1 aerobic training hours per week. Individuals with any known cardiovascular, pulmonary, neuromuscular, or metabolic conditions or those taking any regularly prescribed medications or who were possibly pregnant were excluded from the study. All subjects were encouraged to maintain their current dietary and exercise habits, but were instructed to avoid resistance training of the lower body during the course of the study and aerobic exercise 24 h prior to any visit. Participants were required to complete a health history questionnaire to assess health status and eligibility and gave written acknowledgment of informed consent prior to the start of any data collection protocols. Ethical clearance was given by the university Institutional Review Board (20101111154FB).

### 2.2. Protocol

Each participant was asked to attend a series of five sessions over the course of three weeks; each session was separated by at least 72 h. Participants were instructed to refrain from exercising for at least 48 h prior to the start of each session.

### 2.3. Session One

The first session served the purpose of equipment and procedural familiarization. Following a 5-min warm up on a cycle ergometer at approximately 100 watts, the participants were placed on a Cybex 6000 isokinetic dynamometer (CSMi Solutions, Stoughton, MA, USA) and completed a warm up protocol consisting of five isometric, followed by five concentric, and then five eccentric contractions of the knee extensors at approximately 50% maximal effort [22]. Each participant then completed three maximal isometric, three concentric, and three eccentric contractions. The relative joint angle at the knee was standardized to 120° [23] and time under tension to three seconds during isometric contractions. Angular speed was standardized to 30° s^−1^ and range of motion to 90° (90°–180° at full extension) during concentric and eccentric contractions. Seat and body limb adjustments on the Cybex were recorded during this visit and used in all subsequent visits.

### 2.4. Session Two

The second session was used to determine the pennation angle of the vastus lateralis muscle. sEMG recordings of the vastus lateralis were taken from the dominant leg (based on kicking preference) using a 16-channel linear electrode array (5 × 1 mm dimension, 10 mm inter-electrode distance) with EMG16 data acquisition system (Prima Biomedical and Sport, Treviso, Italy) and EMG_ACQ software (LISiN, Torino, Italy). The signal was differentially amplified with a gain of 66 dB (×2000) and analog filtered using a 4th order Bessel filter (bandwidth = 10–500 Hz). Participants were asked to contract the knee extensors at approximately 50% maximal effort and the innervation zone was identified by the channel with minimal amplitude and phase reversal. The array was set to be on the longitudinal axis of the muscle fibers when the slope of the two lines connecting the sEMG waveforms from channels above and below the innervation zone was equal.

### 2.5. Sessions Three–Five

For each of the remaining sessions, participants completed one of three distinct fatigue-inducing protocols. During each session, participants first completed a warm up consistent with Session 1. A pre-fatigue assessment was then completed to establish non-fatigued measurements (non-fatigued condition), consisting of 3 maximal contractions for each contraction type (isometric, contraction, and eccentric) in random order. During isometric contractions, the relative angle at the knee was standardized to 120° [23] and time under tension to three seconds for each contraction. Angular speed was standardized to 30° s^−1^ and range of motion to 90° (90°–180° at full extension) during concentric and eccentric contractions. This resulted in a 3-s duration consistent with the isometric contractions. A rest period of three seconds was given between all contractions. During the third and fourth sessions, participants completed either a concentric or isometric fatigue protocol in random order. For the fifth session, participants completed an eccentric fatigue protocol. For each protocol in Sessions 3–5, participants completed 30 maximal intermittent contractions (with 3 s of rest between each contraction) of the type specified by the given fatigue protocol. Parameters were standardized and consistent with those during the pre-fatigue assessment (relative knee angle and time under tension for isometric contractions, knee angular velocity, and range of motion for concentric and eccentric contractions). Immediately following, the protocols for the post-fatigue assessment were repeated in identical order as the pre-fatigued assessment to establish fatigued measurements (fatigued condition).

### 2.6. Electromyographic Measurements and Signal Processing

During Sessions 3–5, the sEMG signal was recorded from the vastus lateralis during each contraction of the fatigue protocols as well as the pre- and post-fatigue assessments using a bipolar electrode configuration (circular Ag/AgCl electrodes, diameter = 4 mm, center-to-center inter-electrode distance = 30 mm, sampling frequency = 1000 Hz) (BIOPAC Systems, Inc., Santa Barbara, CA, USA) and differentially amplified with a gain of 60 dB (1000×). Electrodes were placed in line with the longitudinal axis of the vastus lateralis fibers as established during Session 2 with the reference electrode placed over the iliac crest. The sEMG recordings were filtered using a 4th order bandpass Butterworth filter (10–500 Hz) and cropped to retain the middle second of each recording (1.00–2.00 s). This served to avoid the acceleration and deceleration phase for each concentric and eccentric contraction, though for consistency the isometric recordings were cropped as well.

SampEn and DFA α values for each sEMG recording were calculated in Matlab v.2017a (MathWorks, Natick, MA, USA). Briefly, given a sequence of *n* data points, sample entropy is defined as the negative logarithm of the conditional probability that two subsequences of length *m*, which are similar within a tolerance of *r*, remain similar when considering the following data point (*m* + 1). The lower the probability, the more irregular the sequence is said to be. Sample entropy is then given as:

(1) $$SampEn\left(m,r,N\right)=-ln\left(\frac{matches\;of\;length\;m}{matches\;of\;length\;m+1}\right)=-ln\left(\frac{B}{A}\right)$$

where *N* is a time-series of *n* data points, *A* is the number of matches for subsequences of length *m*, defined as the number of similar subsequences within a given tolerance *r*, and *B* is the number of matches for subsequences of length (*m* + 1), both excluding self-matches.

For DFA, a time series *X*(*i*) is first integrated, the integrated series *y*(*k*) is then divided into boxes of equal length *n*. The line of best fit is found using the least squares method to represent the local trend for each box. The integrated series is next detrended by subtracting the local trend *y_n_*(*k*) in each box. The root mean square of the integrated and detrended series is taken to represent the average fluctuation *F*(*n*) for a given box length and is given by:

(2) $$F\left(n\right)=\sqrt{\frac{1}{N}\overset{N}{\underset{k=1}{\sum }}{\left[y\left(k\right)-y_{n}\left(k\right)\right]}^{2}}$$

This process is repeated over the course of all box sizes, α being the slope of the log–log plot of *F*(*n*) against *n*. Detailed explanations of the algorithms are provided by Richman and Moorman [9] (SampEn) and Peng et al. [10] (DFA). Calculations were done using code found on PhysioNet [24]. The embedding dimension (*m*) for SampEn was set to length 2 and tolerance (*r*) to 0.2 multiplied by the standard deviation of the individual sEMG time series [9,25]. Box lengths for DFA were within a minimum value of 4 and maximum value less than 10% of the data length [10,14].

### 2.7. Data Analysis

A factorial MANOVA with repeated measures was used to assess the effects of fatigue status, contraction type, and fatigue protocol on sEMG SampEn and α with top-down applications of repeated-measures ANOVAs and pairwise comparisons as needed. At the univariate level, Greenhouse-Geisser corrections were used when necessary. All statistical analyses were performed using SPSS v.24 (IBM Corp., Armonk, New York, NY, USA). Alpha level was set to 0.05 for all tests.

## 3. Results

### 3.1. Fatigue Status, Contraction Type, and Fatigue Protocol

Results showed a significant multivariate effect from fatigue status (Wilks Λ = 0.214, F(2,10) = 18.34, *p* < 0.001) and contraction type (Wilks Λ = 0.118, F(4,8) = 15.02, *p* = 0.001). Univariate results displayed a significant effect from fatigue status on both SampEn (F(1,11) = 21.36, *p* = 0.001) and α (F(1,11) = 34.75, *p* < 0.001), and from contraction type on both SampEn (F(1.16,12.76) = 8.34, *p* = 0.011) and α (F(1.21,13.25) = 10.26, *p* = 0.005). Pairwise comparisons revealed that SampEn was significantly higher during non-fatigued compared to fatigued conditions (1.57 ± 0.05 vs. 1.46 ± 0.06, respectively, *p* = 0.001), and α was significantly lower during non-fatigued compared to fatigued conditions (1.26 ± 0.05 vs. 1.32 ± 0.05, respectively, *p* < 0.001). With respect to contraction type, SampEn was significantly higher during concentric compared to eccentric (1.58 ± 0.05 vs. 1.47 ± 0.07, respectively, *p* = 0.022) and to isometric (1.58 ± 0.05 vs. 1.50 ± 0.05, respectively, *p* < 0.001) contractions. Furthermore, α was significantly lower during concentric compared to isometric contractions (1.27 ± 0.05 vs. 1.32 ± 0.05, respectively, *p* = 0.001) (Table 1). The results of the repeated-measures ANOVAs indicated that the isometric and concentric fatigue protocols resulted in −17.9% (181.6 ± 33.2 vs. 149.1 ± 26.1 N·m) and −16.6% (188.5 ± 35.4 vs. 157.2 ± 29.1 N∙m) decreases in torque production (collapsed across contraction type), respectively. For the eccentric fatigue protocol, however, there was a significant time (pre-fatigued and fatigued) x contraction type interaction. The follow-up paired sample t-tests indicated that pre-fatigued torque values were greater than fatigued torque values for the isometric (186.9 ± 42.1 vs. 161.3 ± 37.0 N∙m, respectively) and concentric (171.4 ± 25.3 vs. 141.3 ± 24.8 N∙m, respectively) contractions, but not for the eccentric (199.1 ± 61.7 vs. 193.7 ± 48.6 N∙m, respectively) contractions.

### 3.2. Fatigue State * Contraction Type Interaction

**Figure 1:**
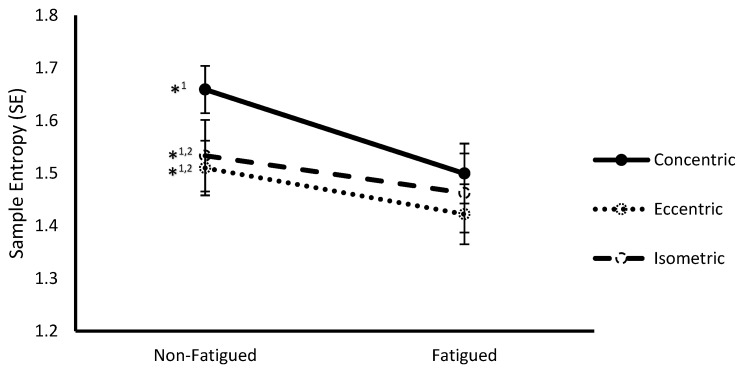
Sample entropy (mean ± SEM) of vastus lateralis surface electromyography (sEMG) signals from non-fatigued to fatigued conditions during concentric, eccentric, and isometric contractions collapsed across fatigue protocol. *^1,2^ indicates a significant difference (*p* < 0.05) with fatigued conditions (1) and concentric contractions (2).

**Figure 2:**
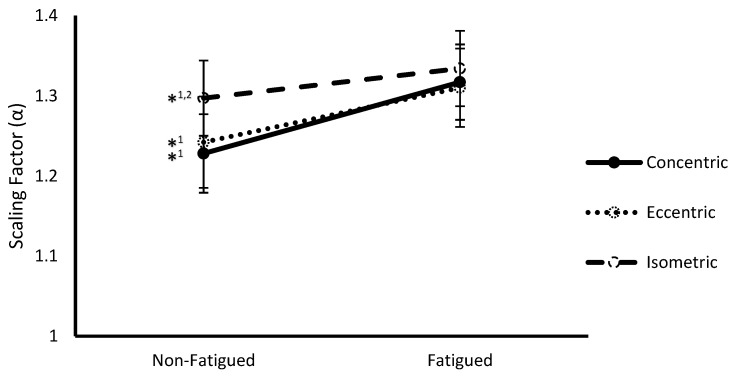
Scaling factor (α) (mean ± SEM) of vastus lateralis sEMG signals from non-fatigued to fatigued conditions during concentric, eccentric, and isometric contractions collapsed across fatigue protocol. *^1,2^ indicates a significant difference (*p* < 0.05) with fatigued conditions (1) and concentric contractions (2).

A significant interaction between fatigue status and contraction type was observed at the multivariate level (Wilks Λ = 0.221, F(4,8) = 7.05, *p* = 0.010). Follow-up ANOVAs revealed significance on both SampEn (F(2,22) = 9.88, *p* = 0.001) (Figure 1) and α (F(2,22) = 6.37, *p* = 0.007) (Figure 2). Post hoc pairwise comparisons revealed that SampEn was significantly higher during non-fatigued compared to fatigued conditions across all contraction types (Concentric: 1.66 ± 0.05 vs. 1.50 ± 0.06, respectively *p* < 0.001; Eccentric: 1.51 ± 0.07 vs. 1.42 ± 0.08, respectively, *p* = 0.008; Isometric: 1.53 ± 0.05 vs. 1.46 ± 0.06, respectively, *p* = 0.016) and α was significantly lower during non-fatigued compared to fatigued conditions for all contraction types (Concentric: 1.23 ± 0.05 vs. 1.32 ± 0.05, respectively, *p* < 0.001; Eccentric: 1.24 ± 0.06 vs. 1.31 ± 0.05, respectively, *p* = 0.001; Isometric: 1.30 ± 0.05 vs. 1.33 ± 0.05, respectively, *p* = 0.011). With respect to contraction type, SampEn was significantly higher for concentric compared to eccentric (1.67 ± 0.05 vs. 1.51 ± 0.07, respectively, *p* = 0.009) and isometric (1.67 ± 0.05 vs. 1.53 ± 0.05, respectively, *p* < 0.001) contractions during non-fatigued conditions. Additionally, α was significantly lower during concentric compared to isometric contractions (1.228 ± 0.05 vs. 1.30 ± 0.05, respectively, *p* = 0.002) during non-fatigued conditions.

### 3.3. Fatigue Protocol * Fatigue Status Interaction

**Figure 3:**
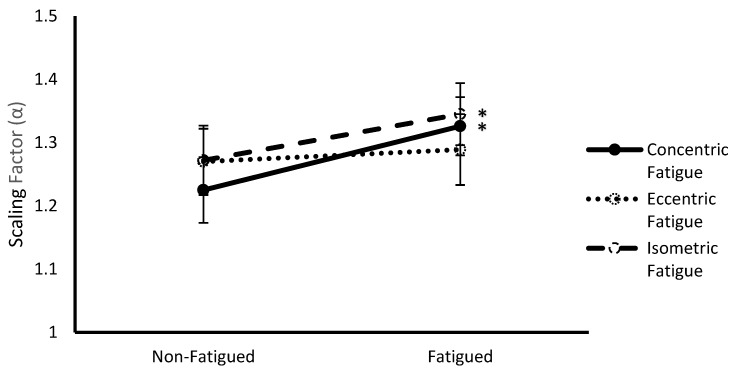
Scaling factor (α) (mean ± SEM) of vastus lateralis sEMG signals from non-fatigued to fatigued conditions during concentric, eccentric, and isometric fatigue protocols collapsed across contraction type. * Indicates a significant difference (*p* < 0.05) with non-fatigued conditions.

A significant multivariate interaction was observed between fatigue protocol and fatigue status (Wilks Λ = 0.334, F(4,8) = 3.98, *p* = 0.046). Follow up ANOVAs revealed significance for α (F(2,22) = 8.85, *p* = 0.002) (Figure 3). Post hoc pairwise comparisons revealed that α was significantly lower during fatigued compared to non-fatigued conditions for concentric (1.23 ± 0.05 vs. 1.33 ± 0.05, respectively, *p* = 0.014) and isometric (1.27 ± 0.06 vs. 1.35 ± 0.05, respectively, *p* = 0.001) fatigue protocols.

## 4. Discussion

The purpose of this study was to investigate the effects of fatigue, contraction type, and fatigue as elicited through different contraction types on sEMG complexity. The present findings showed that complexity was lower during fatigued compared to non-fatigued conditions as indicated by lower SampEn and higher α (closer to 1.5, i.e., brown noise). Furthermore, sEMG recordings during concentric contractions were more complex than during eccentric and isometric contractions as indicated by higher SampEn. Concentric contractions were also found to result in lower α (closer to 1, i.e., pink noise) than isometric contractions, indicative of higher complexity. Considering the multifaceted context of complexity, this exemplifies the need to use multiple statistical measures to assess complexity, each having value in their unique sensitivity for detecting differences between conditions [1]. A significant interaction was observed between fatigue status and contraction type, although importantly it did not confound the main effect interpretations for either fatigue status or contraction type. Specifically, although fatigue resulted in decreased sEMG complexity for all contraction types, the observed decrease was larger for concentric compared to eccentric and isometric contractions. In essence, concentric contractions, although observed to display the highest degree of complexity, were the most affected by fatigue showing the greatest decrease in complexity from non-fatigued to fatigued conditions. Lastly, fatigue induced by concentric and isometric contractions resulted in a loss in complexity as reflected by higher α (more Brownian signals), fatigue induced by eccentric contractions had a comparatively marginal effect in this direction.

SampEn and DFA α have similar (but not identical) interpretations for lower and higher values, respectively. These metrics in turn usually observe an inverse relationship, with changes in a specific direction for one corresponding to opposite changes in the other, and higher values in one corresponding with lower values in the other. While our results showed changes in these metrics in line with the expected relationship (i.e., decreases in SampEn accompanied increases in α), values observed for SampEn and α were similar (~1.5). Previous studies on muscle torque complexity have reported α values similar to those observed for sEMG, though notably different SampEn values (~0.5) [26,27]. Other investigations on sEMG complexity, however, reported SampEn values similar to those observed [4,17]. This may be due to potential differences between sEMG and muscle torque complexity. For example, Suda et al. [17] reported similar findings in low intensity isometric contractions with SampEn values of approximately 1.5–2.0 and 0.05–0.20 for sEMG and muscle torque, respectively. This may also result from the fact that SampEn takes into account a single time scale (embedding dimension) and is highly dependent on this parameter [25], while α takes into account multiple scales. Cashaback et al. [16], utilizing multi-scale entropy, showed that entropy values for biceps brachii sEMG increased with scale until a critical scale at which values plateaued. Further investigation using multi-scale metrics is warranted to investigate this discrepancy.

### 4.1. Effect of Fatigue Status

The present findings showed that fatigue results in a significant loss in sEMG complexity. Consistently, previous research has reported decreases in neuromuscular complexity under fatigued conditions in a variety of neuromuscular outputs such as sEMG [16,17] and muscle torque time series [26,27]. Changes in the complexity of the dynamics of a system occur due to changes in the number of active constituents in a system [1]. Thus, it is thought that neuromuscular complexity is affected by motor unit activation. sEMG complexity has also been shown to increase alongside load and velocity [20,21], both of which correspond to increased motor unit recruitment and discharge rates. Inversely, loss of complexity may result from a decrease in the number of active constituents in a system. It has been suggested that decreases in neuromuscular complexity may occur as a result of motor unit loss or de-recruitment during fatigued conditions [4,17]. Fatigue due to high intensity contractions is known to result in the cessation of specific motor unit firing discharges [28]. Contraction-induced increases in interstitial potassium concentrations result in depolarization of the muscle fiber resting membrane potential in association with fatigue [29]. It is thought that the corresponding decrease in membrane excitability is responsible for the de-recruitment of selected motor units during fatigue [30]. This suggests that the observed fatigue-mediated loss of complexity may be partially attributable to a decrease in motor unit activation during fatigued conditions.

### 4.2. Effect of Contraction Type

The effect of contraction type on complexity has not been previously reported in the literature. The present findings indicated that complexity is significantly greater during concentric compared to both eccentric and isometric contractions. Eccentric contractions display lower levels of motor unit recruitment compared to concentric contractions [31,32]. Thus, lower levels of complexity during eccentric contractions may be due to lower levels of motor unit recruitment. Previous research has suggested the existence of different motor control strategies for concentric and isometric contractions [33,34], though evidence is limited and the details are less clear. The idea of differences between motor control strategies is consistent with that of complexity being task-dependent [35] which relates to the concept of reactive tuning [10], during which the dynamics of a system are temporarily re-structured in a way that facilitates an effective response specific to a given task.

Motor unit activity displays a high degree of interdependence as facilitated through the modulatory effects of interneurons. Specifically, Renshaw cells are known to impose inhibitory effects on motor neurons relative to the activity level of other motor neurons, a form of negative feedback known as recurrent inhibition [36]. Barrue-Belou et al. [37] assessed the degree of recurrent inhibition during maximal voluntary contractions of the plantar flexors under different contraction types, reporting recurrent inhibition to be significantly greater during eccentric compared to concentric and isometric contractions. Higher levels of recurrent inhibition imposed by Renshaw cells would then result in reduced corticospinal excitability. Consistently, prior research reported decreased corticospinal excitability during eccentric contractions in muscles of the lower extremities compared to concentric and isometric contractions [38,39]. Lower corticospinal excitability will result in less motor unit recruitment and thus, partially contribute to observed differences in complexity between concentric and eccentric contractions.

### 4.3. Effect of Fatigue Protocol

To our knowledge, the effect of fatigue on complexity as induced by different contraction types has also not been reported in the literature. The present findings indicated that fatigue induced by concentric and isometric contractions results in less complex sEMG signals compared to eccentric-induced fatigue. Of note, the difference in loss of complexity between contraction types was only reflected by α and not SampEn, which saw a decrease from non-fatigued to fatigued conditions regardless of fatigue protocol (no interaction effect). Considering DFA and SampEn quantify different characteristics of the sEMG signal, this may suggest that the mechanism(s) by which fatigue decreases complexity or the degrees of contributions thereof differ between contraction types. Previous findings have reported neuromuscular functioning to be significantly more resistant to fatigue as induced by eccentric compared to concentric and isometric contractions in the leg extensors [33,40]. This is thought to occur from a higher susceptibility of concentric and isometric contractions to peripheral fatigue relating to higher energy requirement during concentric contractions [40] and restricted blood flow-induced ischemic conditions during isometric contractions [33], which lead to increased intramuscular metabolite concentrations and impaired excitation-contraction coupling. Fatigue-mediated loss of complexity has been shown to occur only above the critical torque threshold [26], above which a metabolic steady state is unattainable [41]. Observed differences in fatigue-mediated loss of complexity between concentric and isometric-induced fatigue compared to eccentric-induced fatigue may be partially related to peripheral factors. Recent work by Pethick et al. [27] suggests that fatigue-mediated loss of complexity in muscle-torque outputs is also in part attributable to central fatigue, although the initiation of events leading to central fatigue is dependent on peripheral factors and is also dependent on the intensity of contractions used to elicit fatigue [6]. Further research is warranted to investigate potential contributions from central and peripheral factors on fatigue-mediated loss of complexity and the effect of contraction-intensity on the degree of these contributions.

## 5. Conclusions

The findings of the present study can be summarized as follows: (1) Fatigue results in a loss of complexity, (2) complexity is contraction-type dependent, and (3) the degree of fatigue-induced loss of complexity depends on the type of contraction used to elicit fatigue. Lower complexity of sEMG signals during fatigued compared to non-fatigued conditions and higher complexity during concentric compared to eccentric contractions may reflect differences in the degree of motor unit activation. Higher complexity during concentric compared to eccentric contractions may also reflect decreased levels of recurrent inhibition. Greater fatigue-induced loss of complexity from concentric and isometric compared to eccentric contractions suggests that loss of complexity is in part attributable to peripheral factors. As complexity has clinical implications with respect to diagnosis and rehabilitation, our findings suggest that contraction type and type of fatigue should be taken into consideration when assessing sEMG complexity.

## Figures and Tables

**Table 1:** Sample entropy and detrended fluctuation analysis scaling factor (α) values (mean ± SE) across all conditions.

Condition		Sample Entropy	Scaling Factor (α)
Fatigue Status	Non-fatigued	1.57 ± 0.05	1.26 ± 0.05
	Fatigued	1.46 ± 0.06 *	1.32 ± 0.05 *
Contraction Type	Concentric	1.58 ± 0.05	1.27 ± 0.05
	Eccentric	1.47 ± 0.07 **	1.28 ± 0.05
	Isometric	1.50 ± 0.05 **	1.32 ± 0.05 **
Fatigue Protocol	Concentric	1.53 ± 0.07	1.28 ± 0.05
	Eccentric	1.50 ± 0.06	1.28 ± 0.05
	Isometric	1.52 ± 0.06	1.31 ± 0.05

* Significant difference (*p* < 0.05) with non-fatigued; ** significant difference (*p* < 0.05) with concentric.
